# Gut Microbiota in Adults with Chronic Widespread Pain: A Systematic Review

**DOI:** 10.3390/diseases13090299

**Published:** 2025-09-09

**Authors:** Pui-Ying Leong, Lin-Hong Shi

**Affiliations:** 1 Division of Allergy, Immunology & Rheumatology, Department of Internal Medicine, Chung Shan Medical University Hospital, Taichung 40201, Taiwan; fiona.leong@gmail.com; 2 School of Medicine, Chung Shan Medical University, Taichung 40201, Taiwan; 3 Institute of Medicine, Chung Shan Medical University, Taichung 40201, Taiwan; 4 Ph.D. Program of Business, Feng Chia University, Taichung 40201, Taiwan; 5 JC School of Public Health and Primary Care, The Prince of Whales Hospital, The Chinese University of Hong Kong, New Territories 999077, Hong Kong

**Keywords:** chronic widespread pain, fibromyalgia, gut microbiota, gastrointestinal microbiota, probiotic, prebiotic

## Abstract

Background/Objectives: Chronic widespread pain (CWP), a key feature of fibromyalgia (FM), has been increasingly associated with gut microbiota alterations, yet the specific changes in microbial composition and the therapeutic potential of probiotics or prebiotics in these patients remain unclear. This systematic review aimed to synthesize current evidence regarding gut microbiota alterations and the effects of microbiota-targeted interventions in individuals with CWP/FM. Methods: A comprehensive search across multiple databases, including PubMed, Web of Science, Cochrane Library, Ovid Embase, Medline, Ovid AMED, and Global Health. These studies were categorized into two primary themes: changes in gut microbiota composition at various taxonomic levels and the therapeutic impact of microbiota-involved treatments in patients with CWP/FM. Results: We finally identified 432 studies, of which 11 met the inclusion criteria. The findings indicate that while alterations in the gut microbiota have been observed in CWP patients, the evidence remains limited and heterogeneous. Conclusions: Preliminary indications suggest a potential role of dysbiosis in the pathophysiology of CWP, but further rigorously designed studies are needed to clarify the therapeutic efficacy of microbiota-based interventions in this patient population.

## 1. Introduction

Chronic widespread musculoskeletal pain (CWP) is a common disorder, affecting some 5–15% of the general population, and presents a sizeable economic burden in terms of disability, work absence, and healthcare costs [1], with a complex etiology. CWP is commonly associated with other physical symptoms such as tiredness, sleep disturbance, and concentration problems [2]. Characterized by CWP and tenderness, sleeping disorders, fatigue, and cognitive dysfunction, fibromyalgia (FM) is the most disabling type of CWP [3], involving a malfunction in pain signaling, resulting in heightened sensitivity (hyperalgesia), pain from normally innocuous stimuli (allodynia), and the spread of pain to larger areas [4], usually linked with other symptoms like stiffness in the muscles and joints, fatigue, poor sleep quality, cognitive problems, anxiety, depression, and irritable intestinal syndrome [5,6]. Growing evidence shows that altered diversity and abundance have been observed in patients with CWP [7,8,9]. Although the microbiota’s association with chronic pain has been fully confirmed [10,11], the exact molecular mechanism is unclear and requires further research.

The complex and multi-dimensional experience of chronic pain syndrome involves various mechanisms, including one of the most noteworthy mechanisms where the gut microbiota’s regulation of pain has been suggested based on the microbiota–gut–brain axis [12]. A diverse array of signaling molecules produced by the gut microbiota—including microbial metabolites, neurotransmitters, and neuromodulators—binds to specific receptors to significantly influence peripheral and central sensitization processes. These processes are key drivers of chronic pain [13]. In the periphery, these microbiota-derived compounds can directly or indirectly enhance the excitability of nociceptive neurons. Within the central nervous system, they can promote neuroinflammation by activating microglia, compromising the blood–brain barrier, and recruiting immune cells, thereby facilitating the establishment and persistence of central sensitization [13,14]. Animal studies have supported these molecular mechanisms in visceral pain [15,16,17,18], neuropathic pain [19,20], inflammatory pain [21,22], and opioid tolerance [23,24]. Human studies have largely focused on visceral pain and found consistent alterations of gut microbiota in individuals with irritable bowel syndrome and abdominal pain [25,26,27,28]. However, data on the possible role of the gut microbiota in the pathophysiology of extra-intestinal diseases like CWP remains scarce.

While the influence of the gut microbiome on the gut–brain axis and systemic health is widely recognized, its specific implications for CWP remain insufficiently synthesized. To our knowledge, a systematic appraisal linking gut microbial composition, derived metabolites, and microbiome-targeted therapeutic interventions specifically to CWP is lacking. This review aims to address this gap by critically evaluating current evidence on gut microbiome and metabolome profiles in CWP, alongside emerging interventional strategies targeting microbiota for pain management.

## 2. Methods

### 2.1. Data Source and Search Strategy

A systematic review was conducted that comprehensively describes the alterations of gut microbiota and microbiota treatments in adult patients with CWP/FM. The protocol has been registered in the International Prospective Register of Systematic Reviews (PROSPERO, CRD42023467275) [29]. The systematic review was conducted according to the latest Preferred Reporting Items for Systematic Reviews and Meta-Analyses (PRISMA) guidelines [30]. A search of Medline/PubMed, Cochrane library, Amed, Global Health, Embase, and Web of Science databases were performed from database inception to 31 December 2024. We combined the keywords “chronic widespread pain”, “fibromyalgia”, and “microbiota” with the Boolean “AND” and “OR” in abovementioned selected databases (detailed in Appendix A).

### 2.2. Eligibility Criteria

Eligibility criteria of included articles were as follows: (1) clinical trial or pilot study or observational study; (2) alteration in the gut microbiota; (2) adult patients (≥18 years old) with CWP and/or FM; (3) and/or include microbiota treatment; and (4) until 31 December 2024.

### 2.3. Data Extraction

Records were managed in Covidence systematic review software (Veritas Health Innovation, Melbourne, Australia; https://app.covidence.org/). Following the removal of duplicate entries, the records were screened to exclude conference abstracts, protocols, review articles, case reports, studies with participants under 18 years of age, and publications that were not pertinent to the review’s aims. The full text of the remaining articles was subsequently obtained and linked to its respective EndNote record. To ensure a comprehensive search, the reference lists of all included studies were also manually examined for any additional relevant publications.

### 2.4. Quality Assessment

The methodological quality of all included articles was independently appraised by two investigators (LHS and PYL). For this assessment, we employed two validated scales: the Physiotherapy Evidence Database (PEDro) Scale for controlled trials and the Methodological Index for Non-Randomized Studies (MINORS) for observational studies. Any discrepancies in assessment between the two reviewers were resolved through discussion to reach a consensus. This approach to quality evaluation was adapted from established methodological frameworks used in prior research [31,32,33].

## 3. Results

**Figure 1:**
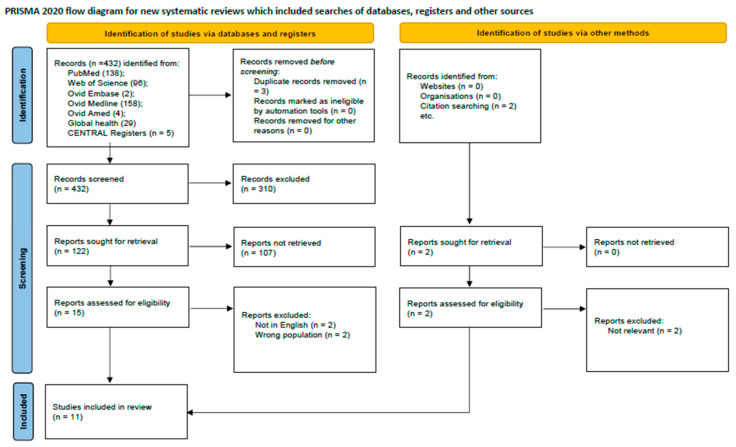
PRISMA flow diagram showing study selection process.

The initial literature search from PubMed, Web of Science, Cochrane library, Ovid Embase, Medline, Ovid Amed, and Global Health databases yielded 432 records, supplemented by two additional records identified from external sources. Following the removal of duplicates and a multi-stage screening process of titles, abstracts, and full-text articles, 11 studies met the eligibility criteria for inclusion. These studies collectively enrolled 20,807 participants. The research landscape was characterized by a high degree of methodological heterogeneity and variable quality among the included articles. The study selection process is detailed in the PRISMA flow diagram (Figure 1), and a summary of included studies is presented in Table 1. Geographically, eight studies were conducted in Europe (Spain, Canada, UK, Denmark) and three in Asia (China, Turkey). The methodological quality for clinical trials and observational studies were acceptable (Table 2 and Table 3).

It would not be possible or appropriate to compare the difference in microbiota composition and measure the effect size of treatment with microbiota in these studies due to heterogeneous study designs (observational studies, case–control studies, and only five RCTs) and differences in patient populations and disease duration. Notwithstanding these limitations, this review summarizes and discusses evidence on the alterations of microbiota and treatment with microbiota in CWP/FM patients.

### 3.1. Alteration of Microbiota in CWP

#### Different Taxonomy Level Alteration

Alterations of microbiota taxonomy levels have been elaborated in Table 4. At diversity level, two studies found that the *alpha diversity* is reduced in FM patients [7,8].

At phylum level, only one study addressed the alteration of microbiota in FM [8], with increased *Firmicutes* and *Bacteroidetes* in these patients, but decreased microbiota at the same time (*Firmicutes*, *Bacteroidetes*, and *Actinobacteria*).

At genus level, four studies observed the alterations of microbiota in FM patients. [8,35,41,45] *Bacteroides* were the common microbiota altered in these studies, with other different genus observed. The abundance of Bacteroides was reduced in FM patients, as were *Bifidobacterium*, *Eubacterium*, and *Clostridium*. However, the abundances of the genera *Dorea*, *Roseburia*, *Alistipes, Roseburia*, *Subdoligranulum*, and *Papillibacter* were increased in this group. Of importance, one large observational study found the causal relationship between alteration of five genus in FM patients; they are *Coprococcus2*, *Eggerthella*, *Lactobacillus*, *FamillyXIIIUCG001*, and *Olsenella* [41].

At family level, two studies reported the relevant results. Only one bacterial family that was found to be diminished in CWP patients (decreased *Ruminococcaceae*) [7], with two families (*Firmicutes* and *Lachnospiraceae*) increased and decreased at the same time. Specifically for FM patients, two bacteria families were observed to be absent in the core microbiota of these patients, *Bifidobacteriaceae* and *Bacteroidales* [8], and other families were decreased. But the *Rikenellaceae* and *Lachnospiraceae* family showed an increased abundance in FM patients [8].

At class level, only one study addressed the alteration in which *Actinobacteria* was decreased in FM patients compared with controls [8].

At the species level, two studies elaborated that different microbiota were altered in FM [45] or CWP patients [7]. The increased *butyriciproducens*, *F. plautii*, *B. desmolans*, *E. tayi*, *E. massiliensis*, *Parabacteroides merdae*, *Akkermansia muciniphila*, and *Clostridium scindens* were observed in FM patients, while it was the reverse for *F. prausnitzii*, *B. uniformis*, *Haemophilus*, *P. copri*, and *Blautia faecis*. In CWP patients [7], massiliensis was shown to be the common one altered between CWP and FM. Increased *odontolyticus* and decreased *excrementihominis*, *obeum*, *formicigenerans*, *splanchnicus*, *ureilytica*, *inulinivorans*, and *Coprococcus comes* were reported.

### 3.2. Metabolic Function of Microbiota in CWP

For FM patients in the study by Minerbi et al. [45], *F. prausnitzii* decreased in FM patients as evident by the presence of circulating short-chain fatty acids (SCFA). Among the 19 species demonstrating significant differential abundance (DA) between FM patients and healthy controls, the level of scientific characterization varied widely. Species found to be depleted in FM patients, such as *F. prausnitzii*, *B. uniformis*, *P. copri*, and *Blautia faecis*, are generally well-documented in the literature [45]. In contrast, species exhibiting increased abundance in patients, including *Intestinimonas butyriciproducens*, *Flavonifractor plautii*, *Butyricoccus desmolans*, and members of the *Eisenbergiella* genus (*E. tayi* and *E. massiliensis*), are less extensively characterized [45]. Moreover, the abundance of the *Bifidobacterium* and *Eubacterium* genera (bacteria participating in the metabolism of neurotransmitters in the host) in FM patients was significantly reduced [8].

Of note, when looking at the association between altered microbiota and clinical functional performance in FM patients, *Bacteroides* spp. was positively correlated with total symptom score on the Fibromyalgia Impact Questionnaire (FIQ) [45].

### 3.3. Treatments with Microbiota in FM

There are only five studies investigating the effects of drugs with microbiota (probiotics or prebiotics) on FM patients (Table 5). A study by Aslan et al. [37] utilized a high-potency probiotic formulation (1 × 10^10 CFU) comprising a multi-strain consortium, including Lactobacillus acidophilus L1, Lactobacillus rhamnosus liobif, Bifidobacterium longum, and Saccharomyces boulardii. This intervention was associated with significant improvements in self-reported pain, sleep quality, and overall quality of life in patients with fibromyalgia. Furthermore, the administration of this probiotic blend was linked to a notable alleviation of depressive and anxiety symptoms, a finding consistent with earlier research in this population [46,47].

The efficacy of gut microbiota-based therapy was assessed using patient-reported outcomes. Two RCTs revealed no statistically significant differences between the intervention and control groups for scores on the visual analogue scale (VAS) or the Fibromyalgia Impact Questionnaire (FIQ) [39,40,43].

Of note, there is only one RCT investigating the potential efficiency of fecal microbiota transplantation (FMT) in FM patients; it seems FMT had improved the clinical symptoms of patients with FM after 6 and 12 months, including widespread pain intensity, anxiety, depression, and sleep. Moreover, certain altered neurotransmitters have been observed with FMT therapy in FM.

In addition, only one prospective cohort study lasting one month has shown the symbiotic intervention where probiotic and prebiotic strains decreased the levels of stress, anxiety as well as depression, and improved quality of life during patients’ daily activities. This intervention improvement was observed to dysregulate the inflammatory and stress responses, particularly in those without a previous chronic fatigue syndrome (CFS) diagnosis.

## 4. Discussion

We aim to systematically evaluate the literature reporting evidence associated with gut microbiota in people with CWP/FM. The review identified broad areas of research including alterations in microbiota composition and its relation to human functions as well as the effects of treatment with microbiota on CWP patients. Based on clinical trials and observational studies with acceptable methodological quality, we found significant alterations of microbiota composition in different taxonomy levels, but the effects of current probiotics or prebiotics are debatable in improving the symptoms of FM patients.

We found few common microbiota across different studies. The alterations of microbiota in different taxonomy levels are quite different across these included studies (Table 5). With rapid developments in microbiota technology over the last two decades, it is not surprising that a diverse set of methodological approaches were employed in the assessment of microbiota compositions, which may account for this observation in this review. We also noted that the diagnosis criteria of FM across included studies is different. For Clos-Garcia et al. [8], the different selections of FM patients may yield different compositions. For CWP patients, although only one study by Freidin et al. [7] reported the composition alterations, it is reasonable that different alterations of microbiota communities were observed in these milder patients, since FM patients who were deemed to be more severe with psychological involvement. However, the studies identified did not provide adequate substance for inclusion, and more future investigations are needed to address this field.

Gut microbiota has been closely linked to metabolic, immunological, and neurological functions in humans. Importantly, in FM patients, an abundance of butyrate producers (*I. butyriciproducens*, *F. plautii*, *B. desmolans*, *E. tayi*, *Parabacteroides merdae*, and *E. massiliensis*) has been detected [48,49,50,51,52,53,54]. While the relative abundance of certain butyrate-producing bacteria was reduced in FM patients, including *F. prausnitzii* and *B. uniformis* [45], an elevated abundance was observed for other microbial taxa with distinct metabolic functions. Notably, FM patients exhibited a higher abundance of *Clostridium scindens* and *B. desmolans*, two species implicated in steroid hormone metabolism through the activity of 20α-hydroxysteroid dehydrogenase, which facilitates the conversion of cortisol to androgens [55,56,57,58,59]. This analysis revealed a higher relative abundance of two specific species in FM patients: Parabacteroides merdae and Akkermansia muciniphila. This finding is intriguing given that these same species were previously identified by Olson et al. as critical mediators of the antiseizure effects of a ketogenic diet [60]. Species with a putative pro-inflammatory role such as *P. copri*, *Bacteroides Uniformis*, and *Haemophilus parainfluenza* were depleted in FM patients [34,61,62]. The alterations of composition observed in our review will be valuable to explore their link to different functions abovementioned.

Although some probiotic species (*Lactobacillus rhamnosus GG*, *L. paracasei*, *L. acidophilus*, and *Bifidobacterium bifidus*) have been used previously to improve functions related to the gut–brain axis [33,39,40], inclusive evidence observed in recent trials preclude the conclusion whether the probiotic or prebiotic treatment is effective in FM patients. However, Roman et al. reported that using probiotic group containing *Lactobacillus acidophilus* or *Lactobacillus Rhamnosus GG ^®^* did not significantly improve depressive or anxiety symptoms when compared to the placebo group [43]. Moreover, another preclinical study [36] reported that probiotics *L. reuteri LR06* or *Bifidobacterium BL5b* had no significant antinociception effects in chronic pain rats.

Unfortunately, there is no study investigating the treatment effect with microbiota on CWP patients, which leaves a huge gap in this field. For CWP patients, an animal study has found that *Lactobacillus rhamnosus GG* (10 billion CFU/150 mL) can attenuate muscle mechanical hyperalgesia in early-life stress-induced widespread muscle pain in adult rats [38]. The different animal findings between FM and CWP may be ascribed to the different species used in animals, but it cannot be ruled out that their effects are different since FM is the severe type of CWP. In addition, whether the therapeutic effect of FMT on the clinical symptoms of FM patients could apply in CWP patients remains uncertain. Future larger cohorts and well-designed RCT are needed to confirm the effect of these therapies on CWP patients.

### 4.1. Interpretation of Findings and Causal Inference

This study identifies several significant alterations in the gut microbiota of individuals with FM/CMP compared to healthy controls, primarily observing a decrease in taxa such as Faecalibacterium prausnitzii, Ruminococcaceae, and Bifidobacteriaceae. The associations reported here do not establish causality; the observed dysbiosis could be a cause, a consequence, or a parallel phenomenon of the pathophysiology of FM/CWP. The functional implications of these taxonomic shifts are informed primarily by preclinical evidence. For instance, we observed a decreased abundance of Faecalibacterium prausnitzii, a species which in vitro and animal studies have shown to be a prominent producer of the anti-inflammatory short-chain fatty acid (SCFA) butyrate [45]. Mechanistic studies suggest that butyrate can modulate immune function by inhibiting histone deacetylase (HDAC) activity and promoting regulatory T-cell differentiation [63,64]. Therefore, it is plausible that the lower levels of F. prausnitzii we observed are associated with a reduced capacity for butyrate production, which may potentially contribute to a pro-inflammatory state that has been hypothesized in FM/CWP. Similarly, the literature from animal models indicates that certain Bifidobacterium species can influence host stress response and cortisol metabolism through the gut–brain axis [65,66]. While this provides a mechanistic rationale for a link between their absence and altered central pain processing, it remains a hypothetical link in humans with FM/CWP. Future intervention studies (e.g., probiotic supplementation) are required to test this causal relationship.

### 4.2. Strengths and Limitations

A significant strength of this review is that this is the first systematic review focusing on CWP patients regarding the alterations in microbiota composition and its relation to human functions as well as the effects of treatment with microbiota. However, methodical differences, heterogeneous study designs, and differences in criteria of patient populations pose limitations to our findings. Moreover, the small number of studies included in investigating microbiota and CWP precluded drawing adequate conclusions. In addition, limitations also included the risk of publication bias and selective reporting given the small number of RCTs and pilot designs. Lastly, although some positive findings have been reported for probiotics/synbiotics and results from one FMT trial and multiple RCTs were negative regarding VAS/FIQ, some limitations of the study designs cannot be neglected.

### 4.3. Future Direction

Future studies should prioritize investigating the functional implications of the sex-dependent microbial signatures identified here, particularly the elevated *Ruminococcus* and *Pseudomonas* in females [35]. While community structure (as reflected in calculated microbiota ratios *Firmicutes*/*Bacteroidetes*, *Bacteroides*/*Prevotella*, and *Roseburia*/*Eubacterium*) may not differ, these specific taxonomic shifts suggest a potential role in mediating the well-documented female predominance in FM prevalence and symptom severity. Targeted culturing and genomic analysis of these gender-specific taxa are needed to elucidate their mechanistic role in sex-biased pain pathways.

## 5. Conclusions

Despite these limitations and the heterogeneity of the studies included, this systematic review suggests that the gut microbiota is altered in different taxonomy levels in CWP patients, and the therapeutic potential of targeted interventions for alleviating fibromyalgia symptoms remains a subject of ongoing scientific discourse. To resolve this, rigorously controlled trials utilizing advanced methodologies, standardized diagnostic criteria, and robust statistical adjustment for confounding variables are critically needed.

## Figures and Tables

**Table 1:** Characteristics of the included studies investigating gut microbiota in subjects with CWP/FM.

Author (Year)	Country	Sample Size	Participants (CWP/FM), n (%)	Study Design	Diagnosis Criteria	Intervention	Controls, n	Outcomes
Çin (2024) [34]	Turkey	53	53 (100)	RCT	NA	Probiotics and prebiotic	18 Probiotics 17 Prebiotic 18 Placebo	Clinical measurements
Calandre 2021 [7]	Canada	110	54 (49.1)	RCT	NA	Probiotic	56 HC	Clinical measurements
Cardona (2021) [35]	Spain	31	16 (51.6)	Pilot RCT	ACR 1990 ACR 2016	Probiotics	15 Placebo	Cognitive and memory measurements
Clos-Garcia (2019) [8]	Spain	159	105 (66)	Case–control	ACR 1990	NA	54 HC	Microbiota Metabolomics
Fang H (2024) [36]	China	45	45 (100)	RCT	ACR 2016	FMT	23 FM	Clinical measurements, Metabolomics
Freidin (2020) [37]	UK	1736	113 (6.5)	Case–control	Modified version of the London Fibromyalgia Epidemiology Study Screening Questionnaire (LFESSQ) in UK	NA	1623 HC	Microbiota Metabolomics
Hinchado (2023) [38]	Spain	15	15 (100)	Cohort	ACR 2016	Synbiotic	7 FM with CFS 8 FM without CFS	Clinical measurements, Inflammatory and stress biomarkers
Minerbi (2019) [39]	Canada	156	77 (49.4)	Case–control	ACObR 2016	NA	11 FC 20 HM 48 UC	Microbiota Metabolites
Ramírez-Tejero (2023) [40]	Spain	41	26 (100)	Pilot descriptive	ACR 2016	NA	15 Male	Microbiota Metabolites
Roman (2018) [41]	Spain	31	16 (51.6)	Pilot RCT	NA	Probiotics	15 Placebo	Cognitive and clinical measurements
Wang (2024) [42]	China	18,430	18,430 (100)	Observational genetic study	NA	NA	NA	Microbiota

RCT: randomized controlled trial; ACR: American College of Rheumatology; HC: health control; FC: family control; FMT: fecal microbiota transplantation; NA: not applicable.

**Table 2:** Scale “Physiotherapy Evidence Database (PEDro)” to analyze the methodological quality of clinical control trial studies.

Author (year)	Specified Selection Criteria	Randomization of Subjects	Allocation Was Concealed	Similar Groups at Baseline	Blinded Subjects	Blinded Therapists	Blinded Assessors	Outcomes Obtained 85%	Treatment or Intervention to Treat	Comparison Between Groups	Points Measure Variability	* Outcome
Aslan et al. (2023) [37]	yes	yes	yes	yes	yes	yes	yes	yes	yes	yes	yes	10
Calandre 2021 [40]	yes	yes	yes	no	yes	yes	yes	yes	yes	yes	yes	9
Cardona et al. (2021) [39]	yes	yes	yes	yes	yes	yes	yes	no	yes	yes	yes	9
Fang H 2024 [42]	yes	yes	yes	yes	yes	yes	yes	yes	yes	yes	yes	10
Roman et al. (2018) [43]	yes	yes	yes	no	yes	yes	yes	yes	yes	yes	yes	9

* Result on the PEDro scale: 9–10 (excellent), 6–8 (good), 4–5 (acceptable), and <4 (poor).

**Table 3:** Scale “Methodological Index for Non-Randomized Studies (MINORS)” to analyze the methodological quality of non-randomized studies.

Items and Author (Year)	Hinchado (2023) [44]	Minerbi et al. (2019) [45]	Clos-Garcia et al. (2019) [8]	Wang (2024) [41]	Ramírez-Tejero et al. (2023) [35]	Freidin et al. (2020) [7]
A clearly stated aim	2	2	2	2	2	2
Inclusion of consecutive patients	2	2	2	2	2	2
Prospective collection of data	2	0	0	0	0	0
Endpoint appropriate to the study aim	2	2	2	2	2	2
Unbiased evaluation of endpoints	2	2	2	2	2	2
Follow-up period appropriate to the major endpoint	2	0	0	0	0	0
Loss to follow-up not exceeding 5%	2	2	2	2	2	2
^ A control group having the gold standard intervention	2	2	2	0	2	2
^ Contemporary groups	2	2	2	2	2	2
^ Baseline equivalence of groups	2	2	2	2	2	2
^ Prospective calculation of the sample size	0	0	0	0	0	0
^ Statistical analyses adapted to the study design	2	2	2	2	2	2
Outcome	22	18	18	16	18	18

^ in the case of non-randomized comparative studies. The items are scored 0 (not reported), 1 (reported but inadequate) or 2 (reported and adequate). The global ideal score being 16 for non-comparative studies and 24 for comparative studies.

**Table 4:** Altered microbiota at different levels in CWP/FM patients.

Author	Study Design	Disease	Level	Increased Microbiota	Decreased Microbiota	Function or Correlation to Human
Clos-Garcia 2019 [8]	Cross-sectional study	FM	diversity		*alpha diversity*	
			phylum	*Firmicutes*	*Firmicutes*	
				*Bacteroidetes*	*Bacteroidetes*	
					*Actinobacteria*	
			genus	*Dorea*	*Bifidobacterium*	
				*Roseburia*	*Eubacterium*	
				*Alistipes*	*Bacteroides*	
				*Roseburia*	*Clostridium*	
				*Subdoligranulum*		
				*Papillibacter*		
			family	*Rikenellaceae*	*Bifidobacteriaceae* (absent)	
				*Lachnospiraceae*	*Bacteroidales* (absent)	
					*Erysipelotichaceae*	
					*Bacteroidales Prevotella*	
					*Eubacterium*	
					*Ruminococcaceae*	
			class		*Actinobacteria*	
Minerbi 2019 [45]	Cross-sectional study	FM	genus	*Intestinimonas*	*Bacteroides*	Bacteroides positively correlated with total symptom score on the Fibromyalgia Impact Questionnaire (FIQ)
				*Flavonifractor*	*Faecalibacterium*	
				*Butyricoccus*		
				*Eisenbergiella*		
				*Enterobacter*		
			species		*F. prausnitzii* (*Faecalibacterium prausnitzii*)	Butyrate producers, antinociceptive as well as anti-inflammatory, enhance the intestinal barrier function, certain short-chain fatty acids (SCFA) producing bacteria
					*B. uniformis* (*Bacteroides uniformis*)	Butyrate producers, certain short-chain fatty acids (SCFA) producing bacteria
					*Haemophilus parainfluenzae*	Butyrate producers, a putative pro-inflammatory role
					*P. copri* (*Prevotella copri*)	Butyrate producers, a putative pro-inflammatory role
					*Blautia faecis*	Butyrate producers
				*Butyriciproducens* (*Intestinimonas butyriciproducens*)		Butyrate producers
				*F. plautii* (*Flavonifractor plautii*)		Butyrate producers
				*B. desmolans* (*Butyricicoccus desmolans*)		Butyrate producers
				*E. tayi* (*Eisenbergiella tayi*)		Butyrate producers
				*E. massiliensis* (*Eisenbergiella massiliensis*)		Butyrate producers
				*Parabacteroides merdae*		Antiepileptic effect
				*Akkermansia muciniphila*		Ketogenic diet effect on seizures
				*Clostridium scindens*		Converting cortisol to androgens by 20α-hydroxysteroid dehydrogenase activity
Freidin 2023 [7]	Cross-sectional study	CWP	diversity		*alpha diversity*	
			family	*Firmicutes*	*Firmicutes*	
				*Lachnospiraceae*	*Lachnospiraceae*	
					*Ruminococcaceae*	
			species	*IOdontolyticus*	*Excrementihominis*	
				*Massiliensis*	*Obeum*	
					*Formicigenerans*	
					*Splanchnicus*	
					*Ureilytica*	
					*Inulinivorans*	
					*Coprococcus comes*	butyrate-producing and anti-inflammatory bacteria
Ramírez-Tejero 2023 [35]	Pilot descriptive study	FM	genus	*Ruminococcus*		
				*Pseudomonas*		
Wang 2023 [41]	Observational genetic study	FM	genus	*Coprococcus2*	*FamilyXIIIUCG001*	
				*Eggerthella*	*Olsenella*	
				*Lactobacillus*		

FM: fibromyalgia; CWP: chronic widespread pain.

**Table 5:** Treatment with microbiota in CWP/FM.

Author	Study Type	Study Design	Patients	Intervention	Contents	Outcome	Summary of Results
Çin 2024 [37]	Human	RCT	FM	Probiotics	4 × 10^10 CFUs per day (Lactobacillus acidophilus L1 (2.9 × 10^9^) and Lactobacillus rhamnosus liobif (2.9 × 10^9^), Bifidobacterium longum (2.9 × 10^9^), and Saccharomyces boulardii (1.3 × 10^9^)	Pain, quality of sleep, quality of life, anxiety, and depressive symptoms	The probiotic group significantly decreased self-reported pain and increased both quality of life and sleep quality, and depressive symptoms and anxiety levels in FM
				Prebiotics	10 g dose inulin per day		
Calandre 2021 [40]	Human	RCT	FM	Probiotic	Multi-strain probiotic, VSL#3^®^	Mean change from the baseline to the endpoint in the composite severity score of the three main gastrointestinal symptoms reported by patients with fibromyalgia (abdominal pain, abdominal bloating, and meteorism)	This study could not demonstrate any beneficial effects of VSL#3^®^ either on the composite score of severity of abdominal pain, bloating, and meteorism or in any of the secondary outcome variables
Cardona 2021 [39]	Human	Pilot RCT	FM	Probiotics	Selected probiotic species (*Lactobacillus rhamnosus GG*, *L. paracasei*, *L. acidophilus*, and Bifidobacterium bifidus), revivification of 6 million germs per capsule, 4 capsules per day	Cognitive functions (memory and attention), a tendency to reduce errors of omission (Go trials) during the Go/No-Go Task	Treatment with a multispecies probiotic produced an improvement in attention by reducing errors on an attention task, but it had no effect on memory. More specifically, a tendency to reduce errors of omission (Go trials) during the Go/No-Go Task was observed after treatment
Fang H 2024 [42]	Human	RCT	FM	FMT	NA	Pain, sleep quality, quality of life, anxiety, depressive symptoms and metabolites	FMT can effectively improve the clinical symptoms of FM. The close relations between the changes in neurotransmitters and FM, certain neurotransmitters may serve as a diagnostic marker or potential target for FM patients
Roman 2018 [43]	Human	Pilot RCT	FM	Probiotics	*Lactobacillus acidophilus* or *Lactobacillus Rhamnosus GG* ^®^	Pain, impact of FMS, quality of life, anxiety and depressive symptoms, computerized cognitive tasks, urinary cortisol	The present results indicate that probiotic treatment did not significantly improve depressive or anxiety symptoms when compared to the placebo group

RCT: randomized controlled trial; FM: fibromyalgia; FMT: fecal microbiota transplantation; NA: not applicable.

## Data Availability

Data is available on request from authors.

## Appendix A. Search Strategy Used for the Medline Database to Identify Literature Reporting on Terms Related to CWP/FM and Gut Microbiota

Search term(s):
1. (“fibromyalgia” [MeSH Terms] OR “fibromyositis” [All Fields] OR “fibrositis” [All Fields] OR “muscular rheumatism” [All Fields])
2. (“chronic pain, widespread” [All Fields] OR “chronic widespread pain” [All Fields])
3. (“microbiota” [MeSH Terms] OR “microbiota” [All Fields] OR “microbiota” [All Fields] OR “microbiota” [All Fields])
4. 1 or 2
5. 3 and 4
6. Limit 10 to English language, Adults
7. Limit 11 to publication date: 1 January 1976 ^ to 31 December 2024
^ the year “fibromyalgia” became the official term for the condition [67].
